# Practical Notes and Formulæ

**Published:** 1880-04-20

**Authors:** 


### PRACTICAL NOTES AND FORMULÆ.

**Excipients.**—*Bread Crumb* is a useful excipient when balsam of Peru is ordered, or when the ingredients are too moist to make a good mass. Two grains of this will make a mass with one grain carbolic acid or one minim creosote. If the bread is crumbly a very little glycerine and tragacanth excipient may be added.

*Calcium Phosphate* possesses in a very remarkable degree the property of giving a greasy substance, such as lard or mercurial ointment, a good pilular consistence, when added in comparatively small quantity.

*Glycerine* alone is not a good excipient, as pills containing it are liable to absorb moisture, and do not take silver well.

*Glycerine* 1 part, with treacle, 3 parts, is an excellent excipient for pil. aloes et mirrhæ.

*Mucilage.*—Pills made with mucilage become very hard in course of time.

*Spirit* should not be used when there is much resin in the pill, and pills made with it should be rolled off very quickly or they will crumble.

*Tragacanth* gives solidity and elasticity to a mass a little too soft, but if too much be added, the pills become so elastic that it is almost impossible to round them with the finisher.

*Tragacanth and Glycerine* is the best excipient for salts and metallic oxides, such as potassium bromide and bismuth subnitrate. The mass should be well kneaded, or more of the excipient will be used than is really necessary.

Tragacanth excipient for pills:

℞ Pulv. tragac.......................................... ʒ j.

Glycerine.............................................. ℥ iv.

Mix.

Or Tragacanth in powder.......................................... ½ oz.

Glycerine and water, of each.......................... 2¼ oz.

Oil of pimento.......................................... 5 drops.

*Wax in small shavings*, with a little powdered soap where not incompatible, is the best excipient for essential oils, carbolic acid, creosote, etc.—*Druggists Circular.*

**Chloroform Vapor in Earache.**—At a recent meeting of the Medical Society of the District of Columbia, Dr. James E. Morgan stated, during a discussion on otitis, that he had often promptly relieved the distressing earache of children by filling the bowl of a common new clay pipe with cotton-wool, upon which he dropped a few drops of chloroform, and inserting the stem carefully into the internal canal, and adjusting his lips over the bowl, blew through the pipe, forcing the chloroform vapor upon the tympanum. Dr. J. Ford Thompson had also accomplished the same relief upon similar principles.—*Boston Journal of Chemistry.*

**Administration of Guaiacum.**—Much attention has lately been paid to the best mode of administering guaiacum. I find that if the alcoholic tincture of the United States pharmacopeia be combined with an equal quantity of liquor potassæ, a perfectly clear solution is obtained, miscible with water in all proportions. The presence of liquor potassæ in the mixture will be advantageous rather than otherwise in most cases in which it is desirable to administer guaiacum. The following formula may prove useful :

℞ Tincture of guaiacum, U. S. P.................... 15 minims.
Solution of potassa.................................. 15 minims.
Glycerine or syrup.................................. 1 drachm.
Cinnamon water to complete........................ 1 ounce.

Of course the dose may be varied at pleasure, but care must be taken to disguise the burning flavor which renders guaiacum somewhat difficult to manage.—*A. H. F. Cameron, in British Med. Journal.*

**For Diphtheria.**—A physician in Illinois writes :

I have used successfully the following for some years for diphtheria:

℞ Sulphite soda.......................................... gr. x.
dissolve in
Warm water.............................................. ℥i.
then add
Salicylic acid............................................ gr. x.

Dose, a teaspoonful every fifteen to thirty minutes (or oftener) to a child of two years. At same time use beef tea, wine, eggs, quinine, etc. I find this an effective anti-zymotic. In bad cases it must be used for some days.—*Boston Journal of Chemistry.*

**Dr. Mussey's Prescription for Dysmenorrhœa.**—During a discussion on dysmenorrhœa, in the Ohio Medical Society, Dr. W. H. Mussey stated that the following formula was very efficacious in all forms of dysmenorrhœa :

℞ Pulveris guaiaci resinæ................................

Terebinthinæ Canadensis ...........................aa ʒi.

Olei sassafras.......................................... ʒii

Alcoholis.................................................. ℥viii.

Macerate for seven days and filter (not strain) ; then add

Hydrargyri chloridi corrosive........................... ℈j.

Dose, from fifteen to thirty drops. It is an old formula and was first used in a prescription in secondary syphilis. As an alterative emmenagogue, it has no equal.—*Journal of Materia Medica.*

**Iodide of Potassium and Calomel.**—It has been frequently observed that the external application of calomel may give rise to severe inflammation of the conjunctiva, if used simultaneously with the administration of iodide of potassium internally. This Dr. Schlæfke explains by the formation of iodate and iodide of mercury, which, in the presence of common salt, or iodide of potassium, are soluble, and act as caustics. He finds that if iodide of potassium be taken twice daily, in half-grain doses, its presence can be constantly detected in the conjunctival sac.—*Med. and Surg. Reporter.*

**The Influence of Varicella on Vaccination.**—On this subject, Dr. A. E. Osborne, of Delaware Co., Pa., writes us:

In your issue for February 14th you mention a case in which varicella had complicated vaccination—the virus laying dormant until the acute disease had run its course. Such cases are certainly interesting, and it might be well to ask of practitioners their experience. I chanced to have a case a couple of years ago, where varicella set in after vaccnation (4–5 days), but failed to influence the virus, and I have a case at present in which varicella showed itself on the sixth or seventh day, and has apparently greatly increased the irritation. Both varicella and vaccination have been severe, and have terminated independently of each other.—*Med. Reporter.*

**Damiana.**—A writer in the Medical Tribune says: "I must declare my convictions unhesitatingly, that damiana acts as a regulator of the sexual system and a remedy for its injuries, in both males and females. It is a help, not to say an actual renovator, of the vital power.- It will remedy the condition which produces nymphomania, but promotes the natural sexual desire. I do not use the alcoholic preparations for obvious reasons. It has not the peculiar influence upon the nervous system, and in certain other respects appears to be actually inert. To this fact, I am certain, much of the dissatisfaction of physicians is to be attributed.

**Injury of Eyes from Chloral.**—Dr. G. H. Felton, of Haverhill, Mass., writes to the Medical Record:

An instance has just come to my knowledge in which two young ladies, sisters, suffered injury to the eyes from the use of chloral. I did not ascertain the amount used, but it was apparently administered in the usual small doses, and continued only a very few days. The eyes in both cases became extremely painful, compelling the patients to remain several days in a dark room, and a permanent weakness remains (after two years), requiring the occasional use of colored glasses, and greatly impairing the usefulness of the eyes. Has chloral ever before been known to produce a similar effect?

**Iodoform and Quina in Ulcers.**—In foul ulcers and sluggish wounds, Dr. Williams reports good results from the following:

℞ Quinia sulphatis........................................ ʒj.

Iodoformi ................................................ gr.xx. M.

Dust the sore several times a day, first washing.—*A Practitioner.*

**Cure of Gleet.**—In his late monogram on gleet, Dr. J. C. O. Will, of London, recommends, as the best and safest of all remedies for the cure of gleet, "the passage once or twice a week of a cold, well-oiled metallic bougie, combined with the internal use of cantharides or ergot."

**Hog-Cholera Medicine.**—The hog-cholera medicine of Wm. A. Denton consists of alum, sulphate of zinc, Venetian red and capsicum.

